# Distinct Epigenetic Domains Separated by a CTCF Bound Insulator between the Tandem Genes, *BLU* and *RASSF1A*

**DOI:** 10.1371/journal.pone.0012847

**Published:** 2010-09-20

**Authors:** Jer-Wei Chang, Han-Shui Hsu, Huey-Juin Ni, Ching-Ting Chuang, Chi-Hui Hsiung, Tim H. Huang, Yi-Ching Wang

**Affiliations:** 1 Department of Pharmacology, College of Medicine, National Cheng Kung University, Tainan, Taiwan; 2 Division of Thoracic Surgery, Taipei Veterans General Hospital, Institute of Emergency and Critical Care Medicine, National Yang-Ming University, Taipei, Taiwan; 3 Department of Life Sciences, National Taiwan Normal University, Taipei, Taiwan; 4 Department of Molecular Virology, Immunology and Medical Genetics, Comprehensive Cancer Center, Ohio State University, Columbus, Ohio, United States of America; CNRS, France

## Abstract

**Background:**

Tumor suppressor gene (TSG) *RASSF1A* and candidate TSG *BLU* are two tandem head-to-tail genes located at 3p21.3. We hypothesized that there may be a concordance on their gene expression and promoter methylation status. If not, then there may be an insulator located between *RASSF1A* and *BLU* genes that provides a barrier activity.

**Methodology/Principal Findings:**

We first identified potential transcriptionally important CpG sites using the methylation-specific oligonucleotide array in relation to mRNA expression of *RASSF1A* and *BLU* genes in primary lung tumors. We demonstrated that E2F1 bound to the potential transcriptionally important CpG sites in *RASSF1A* gene of a normal lung cell line expressing *RASSF1A* transcripts, whereas loss of E2F1 binding to *RASSF1A* in A549 cancer cell line was the result of DNA methylation. Both *RASSF1A* and *BLU* genes had their own potential transcriptionally important CpG regions. However, there was no correlation of methylation status between *RASSF1A* and *BLU*. Using gel shift assay and chromatin immunoprecipitation-PCR (ChIP-PCR), we found that CCCTC-binding factor (CTCF) bound to insulator sequences located between these two genes. Bisulfite sequencing and ChIP-PCR revealed distinct methylation and chromatin boundaries separated by the CTCF binding domains in normal cells, whereas such distinct epigenetic domains were not observed in cancer cells. Note that demethylation reagent and histone deacetylase inhibitor treatments led to CTCF binding and recovery of barrier effect for *RASSF1A* and *BLU* genes in cancer cells.

**Conclusions/Significance:**

Our study dissects the potential transcriptionally important CpG sites for *RASSF1A* and *BLU* genes at the sequence level and demonstrates that CTCF binding to the insulator of *BLU* gene provides a barrier activity within separate epigenetic domains of the juxtaposed *BLU* and *RASSF1A* loci in the 3p21.3 gene cluster region.

## Introduction

Molecular changes in oncogenes and tumor suppressor genes (TSGs) are involved in tumorigenesis [1], [2]. The TSGs are inactivated by genetic and epigenetic abnormalities, including deletion, mutation, promoter hypermethylation, abnormal histone modifications and loss of heterozygosity, [3], [4], [5]. Allelic loss of chromosome 3p21.3 is the most frequent genetic alteration in many sporadic cancers, including hepatocellular carcinomas, gallbladder carcinoma, and breast cancer [6], [7], [8]. In our previous study, the region in 3p21 showed more than 50% loss of heterozygosity in 71 microdissected tumors of primary non-small cell lung cancer (NSCLC) patients compared with their matched normal lung tissues [9]. These data suggested that allelic loss in 3p21.3 and the inactivation of genes in this locus were involved in lung tumorigenesis.

The loss of function of several genes at 3p21.3 such as *Ras association (RalGDS/AF-6) domain family 1 isoform A* (*RASSF1A*) and *zinc finger, MYND-type containing 10* (*ZMYND10/BLU*) has been reported in distinct human carcinomas [7], [8]. *RASSF1A* (NM_007128) is a TSG that encodes a member of the RAS effector family which regulates cell proliferation and apoptosis [10]. RASSF1A and Ras association (RalGDS/AF-6) domain family member 5 (RASSF15/NORE1A) form complexes with mammalian sterile 20 kinases 1 (MST1) or with connector enhancer of KSR 1 (CNK1) and Modulator of Apoptosis-1 (MOAP1) to modulate apoptosis [11], [12], [13]. Song *et al*. reported that RASSF1A inhibited the APC-Cdc20 complex and promoted mitotic arrest at pro-metaphase [14]. However, Liu *et al.* demonstrated that the interaction between RASSF1A and Cdc20 was complex [15]. In addition, RASSF1A and RASSF1C regulated microtubule polymerization and potentially affected the maintenance of genomic stability [16], [17]. Re-expression of *RASSF1A* reduced colony-formation ability in a glioma cell line [18]. Hypermethylation of the *RASSF1A* promoter is frequently found in many human cancers such as lung, breast, kidney, gastric, bladder, neuroblastoma, medulloblastoma, and gliomas tumors [19]. Hypermethylation reduces the transcription and translation of modified genes. Furthermore, *RASSF1A* methylation correlated with poor survival in lung cancer patients [20], [21].

*BLU* (NM_015896) has homology to the MTG/ETO family of transcription factors. Exogenous expression of *BLU* in lung cancer or neuroblastoma cell lines reduced efficiency of colony formation *in vitro* [3]. BLU also functionally suppresses tumor formation in nude mice [22]. Down-regulation of *BLU* RNA expression was observed in nasopharyngeal carcinoma (NPC) cell lines (83%) and NPC biopsies (80%) [22]. *BLU* promoter is commonly hypermethylated in cancers such as glioma, cervical squamous cell carcinomas, NPC, neuroblastoma, and NSCLC [23]. *BLU* is a stress-responsive gene regulated by E2F1, but hypermethylation impairs this response [24].

*RASSF1A* and *BLU* gene loci are located next to each other in the region 3p21.3. Their CpG island hypermethylation and expression status is similar in NSCLC patients [3]. In contrast, other studies report no correlation of hypermethylation of *RASSF1A* and *BLU* promoters in NPC, gliomas, and lung cancer patients [18], [24], [25]. However, the promoters of *RASSF1A* and *BLU* have never been examined in the same series of lung cancer patients for regional signals that modulate protein/RNA expression levels and promoter methylation status. We hypothesized that *RASSF1A* and *BLU* exhibits similar gene expression and promoter methylation status in tumors. If not, then an insulator may exist between *RASSF1A* and *BLU* genes to provide a barrier activity. In the present study, we identified potential transcriptionally important CpG sites using the methylation-specific oligonucleotide (MSO) array method and characterized E2F1 binding to the potential transcriptionally important CpG sites. Second, we demonstrated that CCCTC-binding factor (CTCF) bound to insulators and exhibited barrier activity between *RASSF1A* and *BLU* promoters. These data provided a mechanism for disparate methylation status and expression levels of these two closely located genes. Our data provided evidence that both genes possess their own potential transcriptionally important CpG regions. DNA methylation and transcription of *RASSF1A* and *BLU* promoters are independently controlled within separate epigenetic domains.

## Materials and Methods

### Cell lines and culture conditions

Human normal lung cell lines, IMR90 and MRC5, (American Type Culture Collection, ATCC) and human lung cancer cell lines, A549 and H1299 (ATCC), and CL1-0, CL1-1 and CL1-5 (obtained from Dr. Pan-Chyr Yang, Department of Internal Medicine, National Taiwan University Hospital, Taipei, Taiwan) were cultured in DMEM medium (GIBCO, Grand Island, N.Y.) containing 10% fetal bovine serum (FBS) (BIOCHROM AG, Leonorenstr, Berlin) and 1% penicillin-streptomycin (GIBCO) and incubated at 37°C in 5% CO_2_ atmosphere.

### Clinical sample preparation and DNA/RNA extraction

Tissues were collected after obtaining appropriate institutional review board permission from Taipei Veterans General Hospital and written informed consent from the recruited patients. Surgically resected tumor tissue and corresponding normal tissue were collected from 76 patients diagnosed with primary NSCLC admitted to Veterans General Hospital, Taipei. Of these patients, 55 had adenocarcinomas (AD), 18 had squamous cell carcinomas (SCC), and 3 had large cell carcinomas (LC). Histological classification was determined according to the WHO classification and the tumor-node-metastasis system. Information on the age, sex, and smoking history of the patients was obtained from hospital records. The genomic DNA was prepared using proteinase K digestion and phenol-chloroform extraction. Total RNA was prepared from tumors and normal lung tissues using TRIzol reagent (Invitrogen, Carlsbad, CA). cDNA was synthesized using SuperScript reverse transcriptase (Invitrogen).

### Semiquantitative multiplex reverse transcription-PCR (RT-PCR) assay and quantitative RT-PCR (qRT-PCR)

*RASSF1A* and *BLU* mRNA expression was assayed in a semi-quantitative multiplex RT-PCR and quantitative RT-PCR (qRT-PCR) analysis using the *GAPDH* gene as an internal control. The RT-PCR condition was optimized by adopting the minimal cycle number to avoid a saturation problem. The primer nucleotide sequences, annealing temperature, and PCR cycle number are shown in the Table S1. RT-PCR products were separated on 1.5% agarose gels and visualized under UV illumination. To quantify the relative levels of mRNA expression in the multiplex RT-PCR assay, the value of the *GAPDH* in each reaction was used as the baseline gene expression of that sample and relative value was calculated for the *RASSF1A* and *BLU* genes for each tumor and matched normal samples. Tumor cells that exhibited mRNA expression below normal cells were considered to have an abnormal pattern. qRT-PCR was performed with the KAPA SYBR FAST Universal 2X qPCR Master (Kapa Biosystems, Woburn, MA) in a 20 µl final volume in a Rotor Gene 3000 Real-Time PCR Machine (Qiagen, Valencia, CA). Real-time PCR conditions were as follows: 95°C for 10 min, followed by 40 cycles of 95°C, 20 sec; 60°C, 20 sec; and 72°C, 20 sec. Data were normalized with *GAPDH* expression as internal controls.

### Bisulfite modification and methylation-specific oligonucleotide (MSO) array

**Figure 1:**
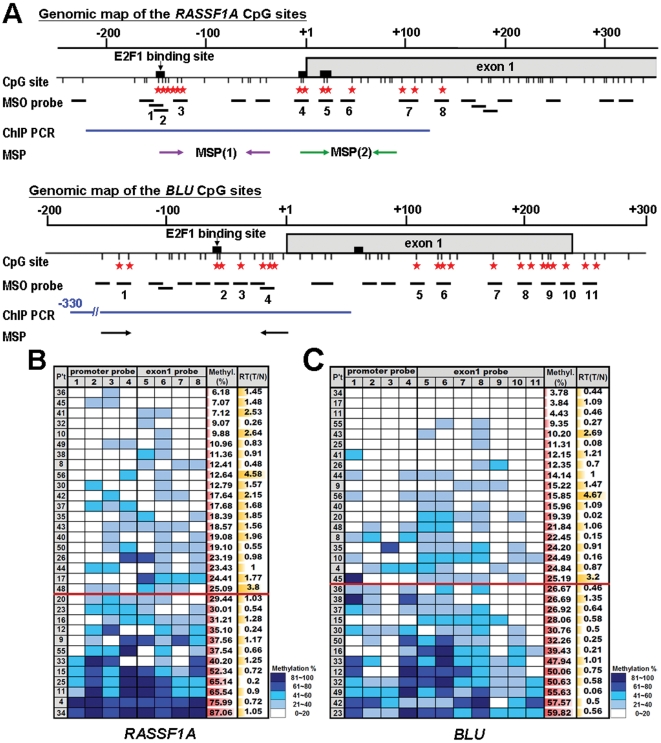
Determination of methylation status of *RASSF1A* and *BLU* CpG sites by MSO array. (**A**) Genomic map of the *RASSF1A* and *BLU* CpG sites detected by MSO were indicated by thin vertical bars. Putative E2F1 binding sites are marked with black boxes. Star symbols indicated the potential transcriptionally important CpG sites. The locations of MSO probe were shown in black horizontal lines. The numbered probes indicated the potential transcriptionally important CpG sites. Blue horizontal lines indicated the regions amplified in the ChIP-PCR assay to detect E2F binding. MSP primers for *RASSF1A* (MSP1 and MSP2) and *BLU* were designed to analyze the methylation status of E2F1 binding sites. (**B**) MSO results are presented for 32 primary lung tumors (left column) in the *RASSF1A* potential transcriptionally important CpG sites (shown in the first row). The patients were then ranked by average methylation percentage determined by MSO method. The average methylation percentage of the potential transcriptionally important CpGs for each patient was listed in the second to the last (right) column. The relative mRNA expression level was calculated for each tumor (T) and matched normal (N) samples and was indicated by RT (T/N) in the right column. The extent of methylation of each CpG was indicated by blue color shown in the lower right corner. The darker shades of blue indicated increasing methylation. (**C**) MSO results were presented for 32 primary lung tumors in *BLU* potential transcriptionally important CpG sites. The heavily methylated and lightly methylated samples were separated by a cut-off line for the *RASSF1A* and *BLU* genes. The cut-off value is determined by the mean methylation percentage of potential transcriptionally important CpG sites.

Tumor DNA from 32 patients (23 were AD, 8 were SCC, and 1 was LC) was analyzed by MSO array. To prepare the methylation-positive DNA, DNA was treated with M. *SssI* methyltransferase (New England Biolabs, Beverly, MA) that methylates all cytosine residues of CpG dinucleotides in the genome. The test samples and *SssI*-treated DNA samples were treated with sodium bisulfite as previously described [26]. Preparation of MSO arrays was carried out essentially as described previously [27]. The oligonucleotides and PCR primers for *RASSF1A* methylation assay were described previously [28]. Twenty sets of paired oligonucleotides were designed to include the promoter and first exon of *BLU* CpG sites. The location of MSO probes for *RASSF1A* and *BLU* was shown in Fig. 1A. For DNA target preparation, bisulfite-treated DNA was amplified from promoter to the first exon regions located in the CpG island using primers listed in Table S1. To control the accuracy and reproducibility of the MSO probes, a series of MSO hybridization were performed with mixed samples containing 100, 66, 33, and 0% *Sss*I-treated methylation-positive DNA [27]. Standardization curves generated with these optimal probe sets were subsequently used to investigate *RASSF1A* and *BLU* methylation in the test tumor DNA samples [27]. Array slide hybridization and wash were described in the previous study [26], [27]. Representative figures were shown in the Fig. S1. The array slides were scanned with a GenePix 4000A scanner (Axon Instruments). MSO data were normalized according to a global ratio in each array image, and the intensity ratio of M/(M+U) (M: methylated probe; U: unmethylated probe) for each probe set was then derived.

### Methylation-specific PCR assay

The methylation status in the E2F1 binding sites of the *RASSF1A* and *BLU* promoters were determined by chemical treatment with sodium bisulfite and subsequent methylation-specific PCR (MSP) analysis. EZ DNA Methylation-Gold™ Kit (Zymo Research, Orange, CA) was used for bisulfite conversion of DNA. MSP analyses used the primers for the –144 to –38 bp (MSP1) and −6 to +94 bp (MSP2) regions in *RASSF1A* promoter, as well as exon1 and −156 to +1 bp region in the *BLU* promoter designing at putative E2F1 binding sites as shown in Fig. 1A. The primer nucleotide sequences, annealing temperature, and PCR cycle number were listed in Table S1. Positive control samples of unmethylated MRC5 normal lung cell DNA and *Sss*I methyltransferase treated methylated DNA were also included for each set of PCR.

### Bisulfite DNA sequencing analysis

The methylation status of the upstream and downstream CTCF regions was examined after bisulfate modification by sequencing analysis. Bisulfite sequencing was conducted for a fragment of chromosome 3 genomic contig (NW_001838877). Primers were designed for CpG sites −60 to +14 (corresponding to nucleotide numbers from −2314 to +137 bp) in which the first CpG site was defined as +1 which corresponded to *RASSF1A* transcriptional start site. Bisulfite-modified DNA was amplified using four pairs of primers: BS-P1, BS-P2, BS-P3, and BS-P4 shown in Table S1. The products were isolated and sub-cloned into the pCR2.1-TOPO vector (Invitrogen), and five individual clones per each sample were sequenced.

### Immunohistochemistry (IHC) assay

Paraffin blocks of tumors were dissected into 5-µm slices and processed using standard deparaffinization and rehydration techniques. Monoclonal antibody for anti-RASSF1A (1∶500; eBioscience, San Diego, CA), and polyclonal antibody for anti-BLU (1∶100; Abcam, Cambridge, UK) were used as the primary antibodies to detect RASSF1A and BLU protein expression, respectively. The evaluation of the immunohistochemistry was conducted without prior knowledge of the clinical and pathologic characteristics of the cases (blinded). RASSF1A and BLU were graded as low or negative expression when tumor cell staining was <20% and <40%, respectively. The surrounding normal stroma and epithelial cells served as an internal positive control for each slide. Normal lung tissue slide from some patients were included to evaluate the IHC results.

### DNA demethylation, histone deacetylation and CTCF transfection

H1299 cells (10^6^) were treated with the demethylating agent 5′-AZA-2′-deoxycytidine (AZA) at 10 µM for 24 hours. The medium was then replaced with medium containing 10 µM AZA and 0.5 µM HDAC inhibitor Trichostatin A (TSA) for 24 hours. After washing with PBS and replacing growth medium, the cells were transfected with pCMV-SPORT6-CTCF expression vector (Open Biosystems, Huntsville, AL) using ExGen 500 *in vitro* transfection reagent (Fermentas, Glen Burnie, Maryland) after AZA and TSA treatments. Samples were incubated for 96 hours and cells were collected for ChIP analysis.

### Chromatin immunoprecipitation (ChIP) and target region ChIP-PCR

Cells were grown on a 100×20-mm culture dish to approximately 80–90% confluency, cross-linked with 1% formaldehyde for 10 min at 37°C, and stopped by the addition of glycine to a final concentration of 0.125 M. Lysates were sonicated using Bioruptor™ system (Diagenode, Liège, Belgium) to shear DNA to lengths between 200 and 800 bp. Subsequent steps were performed with the ChIP assay kit (Upstate Biotechnology, Lake Placid, NY, USA) according to the manufacturer's instructions. ChIP was performed using anti-acetylated lysine 9 of histone H3 (K9Ac) (1∶400; Upstate Biotechnology), anti-trimethylted lysine 27 of histone H3 (K27M) (1∶400; Upstate Biotechnology), anti-E2F1 (1∶500; Active Motif), and anti-CTCF (1∶500; Upstate Biotechnology) antibodies for 16 hours at 4°C. De-crosslinks and purification of immunoprecipitated DNA were performed with the ChIP assay kit (Upstate Biotechnology). The ChIP experiments were repeated three times.

Conditions of PCR analysis with optimized cycle number, primer pairs for *RASSF1A*, *BLU, GAPDH* and *c-Myc* promoters, CTCF binding domains, and regions next to CTCF binding domain were listed in Table S1.

### Electrophoretic mobility shift assay (EMSA)

The CTCF binding sites were predicted from the CTCF binding site database (CTCFBSDB) (http://insulatordb.utmem.edu), and three putative CTCF binding sites were identified in exon 7 (CTCF-1), intron 8 (CTCF-2), and intron 11 (CTCF-3) of *BLU* gene. The probes of CTCF binding sites for the genome sequence of the *BLU* gene were as follows: CTCF-1, +2751 to +2777 bp; CTCF-2, +3555 to +3579 bp; and CTCF-3, +4226 to +4251 bp, and probe sequences were listed in Table S1. A549 cells were transfected with complete CTCF (accession no. BC014267) containing pcDNA3.1 vector for 48 hrs. A549 cell nuclear extracts were prepared and EMSA was performed using biotin end-labeled double-stranded DNA probes prepared by annealing complementary oligonucleotides. The binding reactions were performed using the LightShift Chemiluminescent EMSA Kit (Pierce, Rockford, IL) and conducted according to the manufacturer's protocol. The competition assay utilized a 200-fold excess of unlabeled probe during the EMSA binding reaction. The gel-supershift assay included incubation of the nuclear extracts (12 µg) with the hot probe for 30 min, addition of 2 µg of CTCF polyclonal antibody (Upstate Biotechnology), or the negative control of 2 µg of E2F1 polyclonal antibody (Active Motif) or 2 µg of IgG polyclonal antibody (Upstate Biotechnology) in the binding reaction, and 30 min incubation at RT. Binding mixtures were loaded on a 4% native polyacrylamide TBE gel in 0.5× TBE, and gels were run at 100 V for 2 hour at room temperature. After electrophoresis, the DNA-protein complexes were transferred onto nylon positively charged membranes and detected using chemiluminescence reagent (Pierce).

### Luciferase reporter gene analysis

The E2F1 binding sites were identified using the transcription factor search program PROMO [29]. Promoter sequences of *RASSF1A* were cloned by PCR from IMR90 normal lung cell DNA. Primer pairs used for PCR (−363 to +148 bp) [30] were shown in Table S1. The fragment was cloned into the pGL4 luciferase reporter vector (Promega). Mutations in E2F1 binding sites of *RASSF1A* gene were generated by QuickChange site-directed mutagenesis kit (Stratagene, La Jolla, CA) using the specific primers as: Mut-1, Mut-2 and Mut-3 (Table S1). CL1-1 cell line was co-transfected with 1 µg of the constructs and 2 µg of the pCMV-SPORT6-E2F1 expression vector (Open Biosystems) using ExGen 500 *in vitro* transfection reagent (Fermentas). After 24 hours, the expression of luciferase gene was determined and normalized by Dual-Luciferase reporter assay (Promega).

### Statistical analysis

The statistical analyses of promoter methylation, RNA/protein expression, patient survival and tumor characteristics of 76 patients were performed using SPSS program (SPSS Inc., Headquarters Chicago, IL, USA). Comparisons were made using Chi-square test. The correlation between the MSO methylation level and the mRNA expression level in 32 lung cancer patients was calculated using *t*-test. *P*≤0.05 were considered to be statistically significant.

## Results

### Potential transcriptionally important CpGs in *RASSF1A* and *BLU* gene of lung cancer patients

We examined the association between CpG methylation and RNA expression of *RASSF1A* and *BLU* genes of 32 primary NSCLC tumor samples to identify the CpG sites that were hypermethylated and correlated with low transcriptional level. We used the MSO array to assess methylation profiles of *RASSF1A* and *BLU* CpG island regions in lung cancer patients. Preparation of MSO arrays was carried out essentially as described previously [27]. The 33 CpG sites of *RASSF1A* gene and the 38 CpG sites of *BLU* gene within the CpG island were each examined with a distinct group of 20 oligonucleotide probes. Genomic maps of the *RASSF1A* and *BLU* CpG sites were shown in Fig. 1A. The representative MSO figures are shown in Fig. S1. The average methylation percentage at each CpG region was calculated for all patients. CpG regions that were heavily methylated in all patients were excluded since we could not distinguish their transcriptional importance. The patients were ranked by average methylation percentage in the remaining non-excluded CpG regions. The heavily methylated and lightly methylated patients were separated by a cut-off value which was determined by the mean methylation percentage of potential transcriptionally important CpG sites for the *RASSF1A* and *BLU* genes (Fig. 1B, 1C). To establish the relationship between DNA methylation and gene expression, semi-quantitative RT-PCR with optimized cycle number was conducted on 32 primary lung tumors (Fig. 1B, 1C). Using *t*-test, we found that *RASSF1A* gene exhibited a strikingly significant difference in mRNA expression pattern between the samples with heavily methylated CpG regions and samples with lightly methylated CpG regions (*P* = 0.002), while the expression pattern of *BLU* gene was only modestly different between these heavily and lightly methylated groups (*P* = 0.065). The 8 CpG regions correlating with distinct transcription patterns of *RASSF1A* were located in the proximal promoter and exon 1 region. The 11 CpG regions of *BLU* were located in the proximal promoter and the distal region of exon 1 (Fig. 1A).

### E2F1 binds to the *RASSF1A* promoter at potential transcriptionally important CpGs

**Figure 2:**
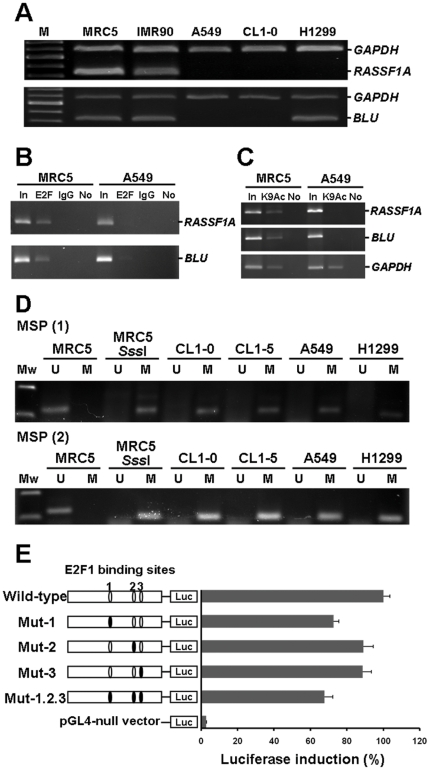
E2F1 bound to the *RASSF1A* promoter at potential transcriptionally important CpGs. (**A**) RT-PCR analysis for *RASSF1A* and *BLU* gene expressions in MRC5 and IMR90 normal cells and three cancer cell lines (A549, CL1-0, and H1299). *GAPDH* was amplified as an internal control. (**B**) ChIP-PCR analysis for E2F1 binding to *RASSF1A* and *BLU* sequences in MRC5 normal cell and A549 cancer cell line. The binding of E2F1 to *RASSF1A* and *BLU* promoters was observed in MRC5 cell line. “In”, total input DNA; “E2F”, DNA-protein complex precipitated by anti-E2F1; “IgG”, DNA-protein complex precipitated by rabbit IgG; and “No”, no antibody. (**C**) ChIP assay with anti-acetylated histone H3 for *RASSF1A*, *BLU* and *GAPDH* loci. “K9Ac”, DNA-protein complex precipitated by anti-acetylated histone H3. (**D**) Methylation status of E2F1 binding sites in *RASSF1A* gene was assessed by MSP in MRC5 normal cell and four cancer cell lines. U: unmethylated gene; M: methylated gene. *Sss*I methyltransferase-treated MRC5 DNA was used as methylation positive control. (**E**) Relative luciferase activities of different E2F1 mutated constructs are shown on the right side as bars in the bar chart, with the structure of each construct shown on the left side. Luc: luciferase gene sequence. E2F1-expression vector was cotransfected with different E2F1 binding sites mutated constructs (mutation sites shown as black circles).

To further investigate gene expression, *RASSF1A* and *BLU* transcripts were analyzed in MRC5 and IMR90 normal lung cell lines and three lung cancer cell lines (A549, CL1-0, and H1299) by semi-quantitative RT-PCR. Both genes were expressed in MRC5 and IMR90, but neither was expressed in A549 and CL1-0 (Fig. 2A). Interestingly, H1299 cell line demonstrated a discordant expression between *BLU* gene and *RASSF1A* gene. Qiu *et al*. demonstrated that the *BLU* promoter was regulated by E2F1 binding, which was impaired by hypermethylation [24]. We thus compared the potential transcriptionally important CpG sites that we had identified with the E2F1 binding site identified by Qiu *et al*. [24]. The data revealed that the CpG sites located at −63/−56 bp matched the consensus E2F1 binding site [24], and indicated E2F1 binding sites were near important CpG sites in the *BLU* gene.

In addition, we identified three putative E2F1-binding sites at −150/−143, −5/+3 and +13/+23 bp within the *RASSF1A* CpG island. They were potential transcriptionally important CpG sites according to the MSO array results **(**upper panel of Fig. 1A). To detect E2F1 binding to these sites, we performed the chromatin immunoprecipitation (ChIP)-PCR assay using the E2F antibody and primers designed to amplify specific fragments from −220 to +116 bp and −330 to +58 bp in *RASSF1A* and *BLU* genes, respectively (Fig. 1A). E2F1 bound to *RASSF1A* and *BLU* genes in the MRC5 normal cell line, but not in A549 tumor cell line (Fig. 2B). To investigate whether exclusion of E2F1 induced the remodeling of chromatin structure in the promoter of *RASSF1A* and *BLU* loci and led to a transcriptionally non-permissive state, MRC5 normal lung cell line and A549 lung cancer cell lines were analyzed by ChIP-PCR. ChIP was performed with antibody against anti-acetylated histone H3 (K9Ac), which recognized acetylated chromatin structure. The *RASSF1A* and *BLU* CpG islands were acetylated in MRC5 and deacetylated in A549. These data suggested that chromatin structure is more compact in the A549 cancer cell line than in the MRC5 normal cell line (Fig. 2C). In addition, loss of E2F1 binding to *RASSF1A* in A549 cancer cell lines correlated with DNA methylation (Fig. 2D). In the luciferase activity assay, mutation in E2F1 binding site at −150/−143 bp (Mut-1), −5/+3 bp (Mut-2), +13/+23 bp (Mut-3), and all E2F1 sites (Mut-1.2.3) within the *RASSF1A* promoter reduced the promoter activity (Fig. 2E). These results demonstrated that potential transcriptionally important CpG sites of *RASSF1A* were bound by E2F1 transcription factor and E2F1 played a role in the activation of the *RASSF1A* gene expression.

### Association between promoter methylation status and expression levels of RASSF1A and BLU

**Figure 3:**
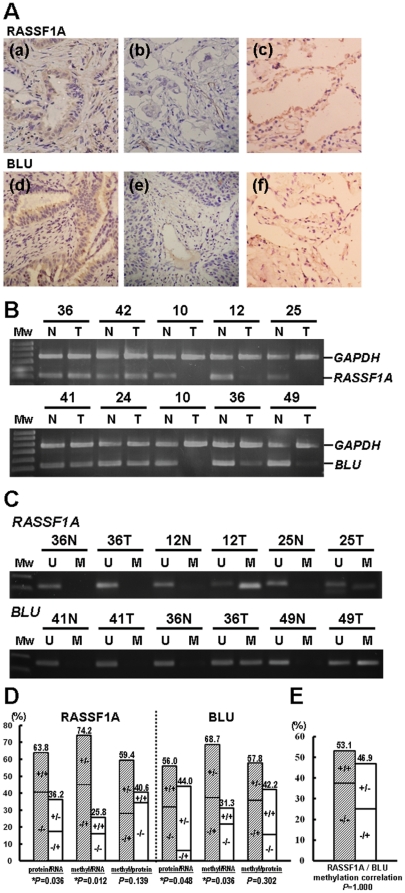
Protein and mRNA expression and promoter methylation assays for *RASSF1A* and *BLU* genes. (**A**) Immunohistochemical analysis of RASSF1A and BLU proteins in paraffin-embedded sections of tumor and normal specimens of representative NSCLC patients. The RASSF1A protein expression status for three samples are marked as panel (a), (b), and (c) and the BLU protein expression status for the other three samples are marked as panel (d), (e), and (f). The cells that exhibited brown color staining in cytoplasm were considered positive for protein expression (patients a and d). Negative immunoreactivity of RASSF1A and BLU was found in two patients (b and e). The histologies in (a) and (d) are adenocarcinomas, (b) and (e) are squamous cell carcinomas, and (c) and (f) are normal lung tissues. Original magnification x200. (**B**) Representative examples of semi-quantitative multiplex RT-PCR analysis of the *RASSF1A* and *BLU* genes in lung cancer patients. *GAPDH* was used as the internal control for the analysis. N and T represent the paired normal and tumor lung cells from the same patient. (**C**) Methylation status of *RASSF1A* and *BLU* genes were assessed by MSP in tumor (T) and matched normal (N) lung samples. (**D**) Concordance analysis between protein expression, mRNA expression, and methylation status of *RASSF1A* and *BLU* genes. Y-axis represents the percentage of cases; X-axis represents the type of comparison. Concordance analysis for RASSF1A and BLU was depicted as percentage. “+” indicated positive protein expression, positive mRNA expression, and DNA hypermethylation, as opposed to “–”, which indicated a negative result. Numbers above the bars indicated the percentage in the total concordant group (gray column) and discordant group (white column). *P* ≤0.05 was considered to be statistically significant and was labeled with *. (**E**) Correlation analysis of methylation status between *RASSF1A* and *BLU*. Numbers above the bars indicated the percentage in the total concordant group (gray column) and discordant group (white column).

To determine whether there was an association between promoter methylation status, protein and mRNA levels of RASSF1A and BLU, 69 NSCLC primary tumors (including 32 patients who had been analyzed by MSO) were assessed by immunohistochemistry (Fig. 3A), multiplex semi-quantitative RT-PCR (Fig. 3B) and methylation-specific PCR (Fig. 3C) assays. Immunohistochemical staining data indicated that 58.0% (40/69) and 37.9% (25/66) of tumors showed an absence or low expression of RASSF1A and BLU proteins, respectively. Semi-quantitative RT-PCR showed the decrease or absence of *RASSF1A* and *BLU* transcripts in 40.6% (28/69) and 50.0% (33/66) tumors, respectively. Methylation-specific PCR (MSP) revealed promoter hypermethylation of *RASSF1A* and *BLU* in 60.6% (40/66) and 48.4% (31/64) tumors, respectively. Cross-tabulation analysis examined the relationship between the methylation status and expression data using the Pearson's χ^2^ test. Aberrantly low protein expression was significantly associated with low mRNA transcript (RASSF1A, *P* = 0.036; BLU, *P* = 0.048). Lack of mRNA expression was significantly associated with promoter methylation (*RASSF1A*, *P* = 0.012; *BLU*, *P* = 0.036) (Fig. 3D). 43 patients were examined for mRNA expression using quantitative RT-PCR (qRT-PCR). The mRNA level detected by qRT-PCR correlated with methylation levels revealed by MSP (Fig. S2) validating the mRNA level detected by the semi-quantitative RT-PCR. Importantly, although *RASSF1A* and *BLU* are two tandem head-to-tail genes located at 3p21.3, the methylation status of the *RASSF1A* gene did not necessarily correlate with the *BLU* gene (Fig. 3E), suggesting that there was no regional effect between these two closely located genes.

### There were CTCF binding sites (insulator DNA sequence elements) between *RASSF1A* and *BLU* genes

**Figure 4:**
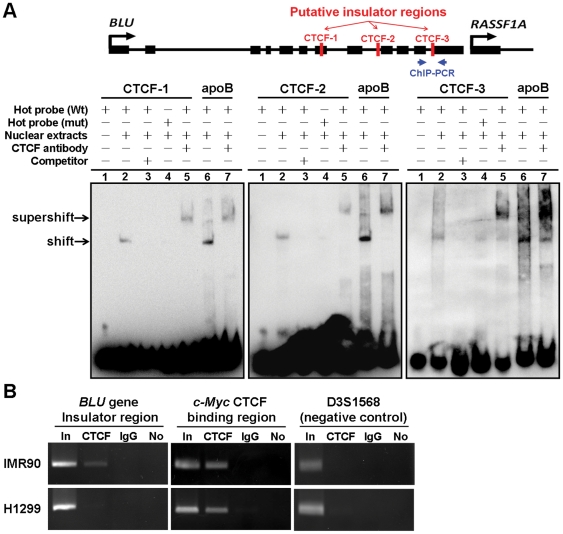
There were three CTCF binding sites between RASSF1A and BLU genes. (**A**) Genomic map on the top shows the position of three putative insulator regions (CTCF-1, CTCF-2, and CTCF-3) between *BLU* and *RASSF1A* gene loci examined in EMSA. The region analyzed for ChIP-PCR assay is as indicated. Bottom figure shows three oligonucleotide probes (CTCF-1, CTCF-2, and CTCF-3) containing putative CTCF binding sites in the *BLU* gene were synthesized and used for EMSA analysis. The biotin-labeled (hot probes) wild-type (Wt) and mutant (mut) oligonucleotide fragments were incubated with A549 nuclear extract and electrophoresed on 4% polyacrylamide gel (lanes 2 and 4). Competition experiments (lane 3) utilized a 200-fold excess of unlabelled wild-type sequences to demonstrate the specificity of each binding reaction. In the presence of anti-CTCF antibody, a supershift complex formed (lane 5). The human *apoB* gene (lanes 6 and 7) was the positive control for EMSA. Arrows indicated the band shift and super-shift of specific protein–DNA complexes. The sequence information for EMSA probes was given in Table S1. (**B**) ChIP-PCR assay for CTCF binding between RASSF1A and *BLU* genes in IMR90 and H1299 cell lines. “In”, total input DNA; “CTCF”, DNA-protein complex pulled down by anti-CTCF; “IgG”, DNA-protein complex pulled down by rabbit IgG; and “No”, no antibody. *c-Myc* served as a positive control for CTCF binding. D3D1568 microsatellite sequence served as a negative control for CTCF binding. Data represented are selected from three independent experiments.

An insulator's function as a barrier against effects from surrounding domains requires association with the CTCF protein [31]. Since we did not find significant correlations of methylation status between *RASSF1A* and *BLU* in the corresponding patients (Fig. 3E), we speculated that there might be CTCF binding sites between *RASSF1A* and *BLU* genes. Using the CTCF binding site database (CTCFBSDB) (http://insulatordb.utmem.edu), putative CTCF binding sites were identified in exon 7 (CTCF-1), intron 8 (CTCF-2), and intron 11 (CTCF-3) of the *BLU* gene. To determine whether CTCF bound to these regions between *RASSF1A* and *BLU* genes, we conducted EMSA with probes carrying wild-type and mutant CTCF binding sites of *BLU* gene. The biotin-labeled probes (hot probes) were incubated with A549 nuclear extract and analyzed on a polyacrylamide gel. The complexes were observed with band shift in wild-type hot probe (lane 2 in Fig. 4A), but showed weak binding in mutant hot probe sample (lane 4 in Fig. 4A). Specificity of binding was determined by competition with an excess of the unlabeled wild-type probe (lane 3 in Fig. 4A). To further corroborate the band shift observations, gel-supershift analysis was performed with a polyclonal antibody of CTCF, and a super-shifted band was detected (lane 5 in Fig. 4A). In addition, CTCF bound to the segment of the human *apoB* gene (lanes 6 and 7 in Fig. 4A) as the positive control [32] and a rabbit IgG binding as the negative control of gel-supershift analysis (Fig. S3) in EMSA. These EMSA results suggested that CTCF formed complexes with three CTCF binding sites in the *BLU* genes. Moreover, ChIP analysis was performed to verify the *in vivo* association of CTCF to the *BLU* gene. *c-Myc* gene was included as a positive control for CTCF binding [33]. ChIP-PCR results indicated that CTCF protein bound to the insulator located between the *RASSF1A* and *BLU* loci in the IMR90 normal cells but not the H1299 (Fig. 4B), A549, and CL1-0 cancer cells (Fig. S4A).

### Distinct methylation and chromatin boundaries separated by the CTCF binding domain in cell lines and lung cancer patients

**Figure 5:**
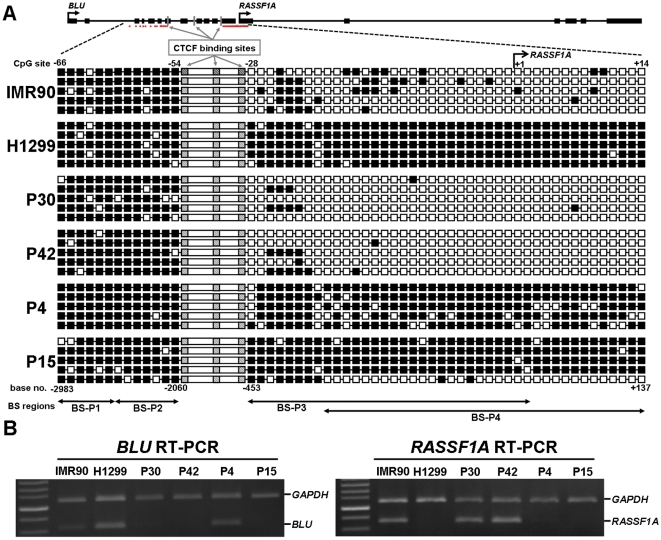
Distinct methylation boundaries separated by the CTCF binding domain. (**A**) Bisulfite sequencing results revealed a distinct methylation boundary separated by the CTCF binding domains in IMR90, H1299 cell line, and four lung cancer patients. CpG sites were designed as −66 to +14 (corresponding to nucleotide numbers from −2314 to +137 bp) in which the first CpG site was defined as +1 and corresponded to the *RASSF1A* transcriptional start site. The red dots below the genomic map indicated the locations of all CpG sites examined. Black box: methylated CpG; write box: unmethylated CpG. CTCF sites were indicated by hatched boxes. The primers used for bisulfite sequencing are shown at the bottom. (**B**) Semi-quantitative multiplex RT-PCR analysis of the *RASSF1A* and *BLU* genes in IMR90, H1299 cell line, and four lung cancer patients which were analyzed by bisulfite sequencing. *GAPDH* was used as the internal control for the analysis.

**Figure 6:**
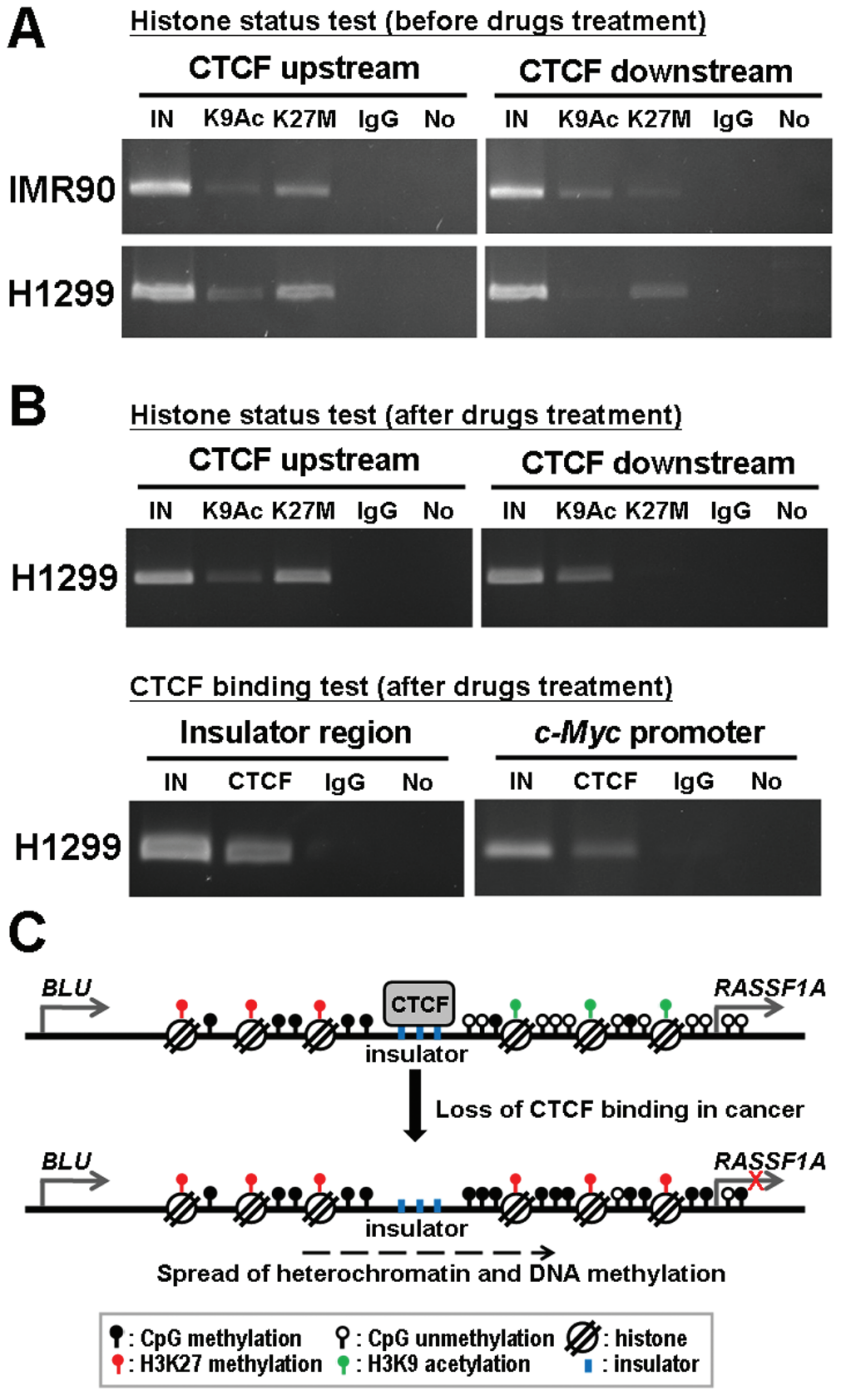
Chromatin structure assays of upstream and downstream for CTCF binding domain. (**A**) Chromatin structure was examined by ChIP-PCR at upstream and downstream of CTCF binding sites using the K9Ac (for open chromatin) and K27M (for compacted chromatin) antibodies in IMR90 and H1299 cells. (**B**) ChIP-PCR for chromatin structure at upstream and downstream of CTCF binding sites (upper panel) and ChIP-PCR for CTCF binding (lower panel) after drug (AZA and TSA) treatments and CTCF exogenous expression in H1299 cell are shown. “In”, total input DNA; “CTCF”, DNA-protein complex precipitated by anti-CTCF; “IgG”, DNA-protein complex precipitated by rabbit IgG; and “No”, no antibody. *c-Myc* served as a positive control. Data represented are selected from three independent experiments. (**C**) Models for CTCF binding to insulator sequence and their effect on hypermethylation of the downstream *RASSF1A* promoter. Top diagram: CTCF binding to insulator sequence and prevention of encroachment of hypermethylation in *RASSF1A* promoter in normal lung cells. Bottom diagram: unbound insulator sequence allowed encroachment of methylation and heterochromatin structure into downstream *RASSF1A* promoter which hindered its transcriptional activity.

If the CTCF binding site within *BLU* gene indeed acted as an insulator between independently controlled methylation domains, patients showing hypermethylation of the *BLU* promoter and lack of methylation in the *RASSF1A* promoter (or *vice versa*) should display distinct methylation boundaries separated by the CTCF binding domains. Therefore, we performed direct bisulfite DNA sequencing of the regions flanking the three CTCF binding sites within *BLU* gene from such NSCLC patients (i.e. patients 30, 42, 4 and 15 selected from Fig. 1B and 1C) as well as IMR90 normal lung cell line and H1299 lung cancer cell line. The upstream and downstream regions for three CTCF binding sites contained 1062 base pairs (spanning CpG site −66 to −54, relative to the *RASSF1A* transcription start site) and 657 base pairs (spanning CpG site −28 to +14) of PCR amplicon, respectively. Bisulfite sequencing revealed a distinct methylation boundary separated by the CTCF binding domains in IMR90 normal lung cell line and patients 30 and 42 (Fig. 5A). Furthermore, the *RASSF1A* expression status examining by semi-quantitative multiplex RT-PCR correlated with *RASSF1A* promoter methylation status in all samples examined (Fig. 5A and 5B). The gene expression status of *BLU* also correlated with promoter methylation status (Fig. 1C and Fig. 5B). In addition, the chromatin structure from each side separated by the presumable insulator sequence was examined for acetylated histone H3 (H3-K9Ac, a histone mark for open chromatin) and tri-methylated histone H3 (H3-K27M, a histone mark for condensed chromatin) in IMR90 and H1299 cell lines. IMR90 cells which had CTCF binding showed a distinct chromatin structure separated by the insulator sequence, whereas H1299 cells without CTCF binding showed a concordant chromatin structure (Fig. 6A). Taken together, these data suggested that the CTCF binding site within *BLU* gene may act as a barrier.

To investigate whether restoration of CTCF binding could change the chromatin structure separated by the presumable insulator sequence in H1299 lung cancer cells, we treated H1299 cells with both the DNA demethylating agent AZA and the HDAC inhibitor TSA. We subsequently exogenously overexpressed CTCF. A ChIP-PCR analysis was performed to detect CTCF binding at 96 hours post-CTCF overexpression. The data indicated that CTCF binding was restored (the lower panel of Fig. 6B). In addition, the chromatin structure of the region located downstream of CTCF binding sites in treated H1299 cells was converted to the pattern seen in normal cell (Fig. 6A and the upper panel of 6B). These data suggested that restoration of CTCF binding to the putative insulator prevented spreading of the repressive chromatin marks past the insulator sequence to downstream *RASSF1A* promoter sequences in the H1299 cancer cells that overexpressed CTCF.

## Discussion

Our study identified the potential transcriptionally important CpG sites in *RASSF1A* and *BLU* genes for the first time, and verified the binding of E2F1 to these CpG sites. In addition, we provided compelling evidence that CTCF binding to an insulator located downstream of the *BLU* gene provides a barrier activity within separate epigenetic domains and regulates the expression of the juxtaposed *BLU* and *RASSF1A* genes.

Transcriptional inactivation by hypermethylation of the promoter regions of TSGs is becoming a common phenomenon in tumorigenesis. The MSO array can be applied to map methylation CpG sites within the CpG island of a candidate gene in normal or tumor DNA. This method was used for mapping methylation changes at individual CpG site in CpG island loci of *ERa*, *p16INK4A*, and *hMLH1* genes in different cancers [26], [34], [35]. In this paper, we combined MSO array methylation data and RNA expression status from lung cancer patients to identify potential transcriptionally important CpG sites of *RASSF1A* and *BLU* promoters (Fig. 1). When these potential transcriptionally important CpG sites were hypermethylated, transcription factors such as E2F failed to bind to the promoter and the hypermethylation hindered gene transcription and expression (Fig. 2). However, some of patients showed discordance between methylation level and transcription level of potential transcriptionally important CpG regions. These discordant results may be due to the presence of several distinct tumor subpopulations, one of which has methylation and does not express the mRNA, the others having no methylation and expressing the mRNA. Alternatively, methylation of only one allele in these tumors may result in the positive mRNA expression observed. Future studies that analyze the relationship of methylation of each CpG site with expression levels by luciferase activity assay can further decipher regulatory mechanisms.

E2F1 transcription factor failed to bind to the recognition sequence that contained methylated CpG dinucleotides [36]. In our study, we demonstrated for the first time that E2F1 bound to the potential transcriptionally important CpG sites of *RASSF1A* promoter. We also confirmed that hypermethylation at the CpGs located within the E2F1 binding sites blocked E2F1 binding and suppressed *RASSF1A* expression in A549 cancer cell line (Fig. 2D). In addition, exclusion of E2F1 binding may result in the remodeling of chromatin in the region into a transcriptionally non-permissive state [37], in agreement with the compact chromatin structure of *RASSF1A* and *BLU* promoters in the A549 cell (Fig. 2C). However, site-directed mutagenesis of E2F sites of the *RASSF1A* promoter only partially reduced its promoter activity, which suggested that other activators such as SP1 may be involved in the transcriptional activation of *RASSF1A* gene [30].

Many studies demonstrate hypermethylation of the *RASSF1A* and *BLU* gene promoters and low mRNA expression in various cancers [3], [10], [24]. However, most studies show no correlation between low *RASSF1A* and *BLU* mRNA expression in cancers. Our study demonstrated that there were no correlations of methylation status (Fig. 3E) or expression status (Fig. S5) between these two genes in NSCLC patients. These results suggested that there was no regional effect between these two head-to-tail located genes. Insulators are DNA elements that prevent inappropriate interactions between neighboring chromatin domains, and CTCF is a major insulator binding protein in the vertebrates that mediate the insulator activity. CTCF is a critical transcription factor which is involved in transcription activation and repression by binding the chromatin insulators [38]. In addition, CTCF also has barrier function to prevent spreading of heterochromatin structure and DNA methylation which plays a critical role in maintaining methylation-free zones [39], [40]. In recent study, Witcher and Emerson identified CTCF binding to a region (∼1.8 kb) upstream of the *RASSF1A* promoter [33], which was further refined to exon 7, intron 8, and intron 11 of *BLU* gene by our study. Our bisulfite sequencing and ChIP-PCR results revealed distinct methylation and chromatin structure boundaries separated by the CTCF binding domains which are located at the 3′ end of *BLU* gene in IMR90 normal cell line (Fig. 5A and 6A). Furthermore, CTCF unbound insulator sequence allowed encroachment of methylation and heterochromatin structure into downstream *RASSF1A* promoter which hindered its transcriptional activity in H1299 cancer cell (Fig. 4B and Fig. 5). However, the *BLU* gene expression does not correlate with CTCF binding status (Fig. 5), because the transcriptional activity of this gene is regulated by promoter methylation (Fig. 2A and Fig. S4). These data suggested that CTCF binding to the insulator prevented spreading of DNA methylation and post-translational modifications of chromatin from the *BLU* locus to the *RASSF1A* gene promoter. In addition, H1299 cancer cell treated with demethylating reagent (AZA), histone deacetylase inhibitor (TSA) and overexpression of CTCF led to restoration of CTCF binding as well as a change of chromatin structure separated by the presumable insulator sequence between *RASSF1A* and *BLU* loci (Fig. 6B). These results indicated that the boundary function is restored in H1299 cancer cell.

It was interesting that AZA and TSA treatment of H1299 cancer cell changed the chromatin structure in downstream sequences between *BLU* and *RASSF1A* genes, but not upstream of the CTCF binding sites (Fig. 6A and the upper panel of 6B). Recently, MYC protein recruited the PRC2/EZH2/DNMT3B complex to bind to the *RASSF1A* promoter, which led to *RASSF1A* gene silencing by promoter CpGs hypermethylation and condensed chromatin in cancer cell [41]. EZH2, a subunit of Polycomb repressive complex 2 (PRC2), is a histone methyltransferase that methylates H3K27 [42]. PRC2-mediated transcriptional silencing has been impeded by the histone deacetylase inhibitor TSA [43]. Our results that AZA and TSA treated H1299 cancer cell changed the chromatin structure nearby the *RASSF1A* promoter, but not upstream of the CTCF binding sites between *RASSF1A* and *BLU* loci supported their mechanisms [41]–[43].

In summary, we used MSO array and mRNA expression data to identify the potential transcriptionally important CpG sites of *RASSF1A* or *BLU* gene promoter in NSCLC patients. We also discovered that the potential transcriptionally important CpG sites of *RASSF1A* were bound by E2F1 transcription factor. We provided compelling evidence that there are CTCF binding sites between *RASSF1A* and *BLU* genes, and occupied CTCF sites prevent the encroachment of methylation and repressive chromatin from the neighboring gene. Although *RASSF1A* and *BLU* genes are tandem head-to-tail genes located at 3p21.3 that contains a 171 bp intervening sequence, CTCF binding to the insulator sequence located at the 3′ end of *BLU* gene, but upstream of the *RASSF1A* promoter, may provide barrier activity between these two loci (Fig. 6C). Further refined mapping of CTCF binding region by ChIP-sequencing and identification of other proteins involved in insulator function are worthy of investigation.

## Supporting Information

Figure S1 Representative MSO array figures. (A) Standardization curve for MSO assays. Upper panel, the series of MSO hybridization were performed with mixed samples containing 0, 33, 66, and 100% of in vitro-methylated DNA and amplified by PCR for the RASSF1A CpG island. The Cy5 fluorescent dye was added to the 3′ end of amplified fragments, and signals of the methylated (M) and unmethylated (U) probes for RASSF1A CpG region #4 were shown, which reflected the indicated percentage of methylation. Lower panel, standard curve for measuring methylation level for RASSF1A CpG region #4 was shown. The intensity ratios (Y-axis) represented signal intensities of M/M+U. The linear distribution showed that measurements of the different mixtures were easily distinguished and used to determine the methylation level in the same CpG region for the patient samples. (B) Hybridization of three lung tumor samples to MSO microarray and images corresponding to the RASSF1A CpG region #4. Patient number 45 showed 0% methylation, but patient #16 and #9 displayed 58.4% and 79.3% methylation, respectively, based on the intensity ratios calculated from the standard curve.
(0.09 MB DOC)

Figure S2 Concordance analysis between mRNA expression (qRT-PCR) and methylation status (MSP) of RASSF1A and BLU genes. Y-axis represents the percentage of cases; X-axis represents the type of comparison. “+” indicates positive mRNA expression and DNA hypermethylation, as opposed to “−”, which indicates a negative result. Numbers above the bars indicate the percentage in the total concordant group (gray column) and discordant group (white column). P values are as indicated.
(0.06 MB DOC)

Figure S3 Three CTCF binding sites between RASSF1A and BLU genes by EMSA analysis. Three oligonucleotide probes (1, 2, and 3) containing putative CTCF binding sites in the BLU gene were synthesized and used for EMSA analysis. The biotin-labeled (hot probes) wild-type (Wt) oligonucleotide fragments were incubated with A549 nuclear extract and electrophoresed on 4% polyacrylamide gel. In the presence of anti-E2F1 antibody (lanes 1 to 3) and anti-IgG antibody (lanes 4 to 6), no super-shift complex was formed. The human apoB gene (lane 7) was the positive control for CTCF binding. Arrows indicated the band shift of specific protein-DNA complexes. The sequence information for EMSA probes is given in Table S1.
(0.17 MB DOC)

Figure S4 CTCF binding assay and promoter methylation assay of RASSF1A and BLU genes in A549 and CL1-0 cancer cell lines. (A) ChIP-PCR assay for CTCF binding between RASSF1A and BLU genes in A549 and CL1-0 cell lines. “In”, total input DNA; “CTCF”, DNA-protein complex pulled down by anti-CTCF; “IgG”, DNA-protein complex pulled down with rabbit IgG; and “No”, no antibody. c-Myc served as a positive control for CTCF binding. D3D1568 microsatellite sequence served as a negative control for CTCF binding. (B) Methylation status of RASSF1A and BLU genes were assessed by MSP in MRC5 normal cell, A549 and CL1-0 cancer cell lines. U: unmethylated gene; M: methylated gene. SssI methyltransferase-treated MRC5 DNA was used as methylation positive control.
(0.16 MB DOC)

Figure S5 Correlation analysis of mRNA and protein expression between RASSF1A and BLU. Y-axis: the percentage of cases; X-axis: the type of comparison. “+” indicated positive mRNA expression and protein expression, as opposed to “−”, which indicated a negative result. Numbers above the bars indicate the percentage in the total concordant group (gray column) and discordant group (white column). P values were compared results of RASSF1A with BLU.
(0.05 MB DOC)

Table S1 Primer sequences, annealing temperature, and number of cycles in PCR reaction.
(0.11 MB DOC)

## References

[1] Fong KM, Sekido Y, Minna JD (1999). Molecular pathogenesis of lung cancer.. J Thorac Cardiovasc Surg.

[2] Sekido Y, Fong KM, Minna JD (1998). Progress in understanding the molecular pathogenesis of human lung cancer.. Biochim Biophys Acta.

[3] Agathanggelou A, Dallol A, Zochbauer-Muller S, Morrissey C, Honorio S (2003). Epigenetic inactivation of the candidate 3p21.3 suppressor gene BLU in human cancers.. Oncogene.

[4] Thiagalingam S, Foy RL, Cheng KH, Lee HJ, Thiagalingam A (2002). Loss of heterozygosity as a predictor to map tumor suppressor genes in cancer: molecular basis of its occurrence.. Curr Opin Oncol.

[5] Wang YC, Chen CY, Chen SK, Cherng SH, Ho WL (1998). High frequency of deletion mutations in p53 gene from squamous cell lung cancer patients in Taiwan.. Cancer Res.

[6] Martinez A, Walker RA, Shaw JA, Dearing SJ, Maher ER (2001). Chromosome 3p allele loss in early invasive breast cancer: detailed mapping and association with clinicopathological features.. Mol Pathol.

[7] Riquelme E, Tang M, Baez S, Diaz A, Pruyas M (2007). Frequent epigenetic inactivation of chromosome 3p candidate tumor suppressor genes in gallbladder carcinoma.. Cancer Lett.

[8] Tischoff I, Markwarth A, Witzigmann H, Uhlmann D, Hauss J (2005). Allele loss and epigenetic inactivation of 3p21.3 in malignant liver tumors.. Int J Cancer.

[9] Tseng RC, Chang JW, Hsien FJ, Chang YH, Hsiao CF (2005). Genomewide loss of heterozygosity and its clinical associations in non small cell lung cancer.. Int J Cancer.

[10] Donninger H, Vos MD, Clark GJ (2007). The RASSF1A tumor suppressor.. J Cell Sci.

[11] Ortiz-Vega S, Khokhlatchev A, Nedwidek M, Zhang XF, Dammann R (2002). The putative tumor suppressor RASSF1A homodimerizes and heterodimerizes with the Ras-GTP binding protein Nore1.. Oncogene.

[12] Rabizadeh S, Xavier RJ, Ishiguro K, Bernabeortiz J, Lopez-Ilasaca M (2004). The scaffold protein CNK1 interacts with the tumor suppressor RASSF1A and augments RASSF1A-induced cell death.. J Biol Chem.

[13] Vos MD, Dallol A, Eckfeld K, Allen NP, Donninger H (2006). The RASSF1A tumor suppressor activates Bax via MOAP-1.. J Biol Chem.

[14] Song MS, Song SJ, Ayad NG, Chang JS, Lee JH (2004). The tumour suppressor RASSF1A regulates mitosis by inhibiting the APC-Cdc20 complex.. Nat Cell Biol.

[15] Liu L, Baier K, Dammann R, Pfeifer GP (2007). The tumor suppressor RASSF1A does not interact with Cdc20, an activator of the anaphase-promoting complex.. Cell Cycle.

[16] Dallol A, Agathanggelou A, Tommasi S, Pfeifer GP, Maher ER (2005). Involvement of the RASSF1A tumor suppressor gene in controlling cell migration.. Cancer Res.

[17] Vos MD, Martinez A, Elam C, Dallol A, Taylor BJ (2004). A role for the RASSF1A tumor suppressor in the regulation of tubulin polymerization and genomic stability.. Cancer Res.

[18] Hesson L, Bieche I, Krex D, Criniere E, Hoang-Xuan K (2004). Frequent epigenetic inactivation of RASSF1A and BLU genes located within the critical 3p21.3 region in gliomas.. Oncogene.

[19] Agathanggelou A, Cooper WN, Latif F (2005). Role of the Ras-association domain family 1 tumor suppressor gene in human cancers.. Cancer Res.

[20] Burbee DG, Forgacs E, Zochbauer-Muller S, Shivakumar L, Fong K (2001). Epigenetic inactivation of RASSF1A in lung and breast cancers and malignant phenotype suppression.. J Natl Cancer Inst.

[21] Kim DH, Kim JS, Ji YI, Shim YM, Kim H (2003). Hypermethylation of RASSF1A promoter is associated with the age at starting smoking and a poor prognosis in primary non-small cell lung cancer.. Cancer Res.

[22] Yau WL, Lung HL, Zabarovsky ER, Lerman MI, Sham JS (2006). Functional studies of the chromosome 3p21.3 candidate tumor suppressor gene BLU/ZMYND10 in nasopharyngeal carcinoma.. Int J Cancer.

[23] Hesson LB, Cooper WN, Latif F (2007). Evaluation of the 3p21.3 tumour-suppressor gene cluster.. Oncogene.

[24] Qiu GH, Tan LK, Loh KS, Lim CY, Srivastava G (2004). The candidate tumor suppressor gene BLU, located at the commonly deleted region 3p21.3, is an E2F-regulated, stress-responsive gene and inactivated by both epigenetic and genetic mechanisms in nasopharyngeal carcinoma.. Oncogene.

[25] Marsit CJ, Kim DH, Liu M, Hinds PW, Wiencke JK (2005). Hypermethylation of RASSF1A and BLU tumor suppressor genes in non-small cell lung cancer: implications for tobacco smoking during adolescence.. Int J Cancer.

[26] Gitan RS, Shi H, Chen CM, Yan PS, Huang TH (2002). Methylation-specific oligonucleotide microarray: a new potential for high-throughput methylation analysis.. Genome Res.

[27] Yan PS, Wei SH, Huang TH (2004). Methylation-specific oligonucleotide microarray.. Methods Mol Biol.

[28] Yan PS, Shi H, Rahmatpanah F, Hsiau TH, Hsiau AH (2003). Differential distribution of DNA methylation within the RASSF1A CpG island in breast cancer.. Cancer Res.

[29] Messeguer X, Escudero R, Farre D, Nunez O, Martinez J (2002). PROMO: detection of known transcription regulatory elements using species-tailored searches.. Bioinformatics.

[30] Strunnikova M, Schagdarsurengin U, Kehlen A, Garbe JC, Stampfer MR (2005). Chromatin inactivation precedes de novo DNA methylation during the progressive epigenetic silencing of the RASSF1A promoter.. Mol Cell Biol.

[31] Bell AC, West AG, Felsenfeld G (2001). Insulators and boundaries: versatile regulatory elements in the eukaryotic.. Science.

[32] Antes TJ, Namciu SJ, Fournier RE, Levy-Wilson B (2001). The 5′ boundary of the human apolipoprotein B chromatin domain in intestinal cells.. Biochemistry.

[33] Witcher M, Emerson BM (2009). Epigenetic silencing of the p16(INK4a) tumor suppressor is associated with loss of CTCF binding and a chromatin boundary.. Mol Cell.

[34] Mund C, Beier V, Bewerunge P, Dahms M, Lyko F (2005). Array-based analysis of genomic DNA methylation patterns of the tumour suppressor gene p16INK4A promoter in colon carcinoma cell lines.. Nucleic Acids Res.

[35] Zhang D, Bai Y, Ge Q, Qiao Y, Wang Y (2006). Microarray-based molecular margin methylation pattern analysis in colorectal carcinoma.. Anal Biochem.

[36] Campanero MR, Armstrong MI, Flemington EK (2000). CpG methylation as a mechanism for the regulation of E2F activity.. Proc Natl Acad Sci U S A.

[37] Zhang B, Chambers KJ, Leprince D, Faller DV, Wang S (2009). Requirement for chromatin-remodeling complex in novel tumor suppressor HIC1-mediated transcriptional repression and growth control.. Oncogene.

[38] Gaszner M, Felsenfeld G (2006). Insulators: exploiting transcriptional and epigenetic mechanisms.. Nat Rev Genet.

[39] Filippova GN (2008). Genetics and epigenetics of the multifunctional protein CTCF.. Curr Top Dev Biol.

[40] Mukhopadhyay R, Yu W, Whitehead J, Xu J, Lezcano M (2004). The binding sites for the chromatin insulator protein CTCF map to DNA methylation-free domains genome-wide.. Genome Res.

[41] Palakurthy RK, Wajapeyee N, Santra MK, Gazin C, Lin L (2009). Epigenetic silencing of the RASSF1A tumor suppressor gene through HOXB3-mediated induction of DNMT3B expression.. Mol Cell.

[42] Muller J, Hart CM, Francis NJ, Vargas ML, Sengupta A (2002). Histone methyltransferase activity of a Drosophila Polycomb group repressor complex.. Cell.

[43] Varambally S, Dhanasekaran SM, Zhou M, Barrette TR, Kumar-Sinha C (2002). The polycomb group protein EZH2 is involved in progression of prostate cancer.. Nature.

