# Supplementary material for: Distinct Epigenetic Domains Separated by a CTCF Bound Insulator between the Tandem Genes, BLU and RASSF1A
Source: PLoS One. 2010 Sep 20;5(9):e12847. doi: 10.1371/journal.pone.0012847 (PMC2942851; doi:10.1371/journal.pone.0012847)
Supplement: Figure S1 — Representative MSO array figures. (A) Standardization curve for MSO assays. Upper panel, the series of MSO hybridization were performed with mixed samples containing 0, 33, 66, and 100% of in vitro-methylated DNA and amplified by PCR for the RASSF1A CpG island. The Cy5 fluorescent dye was added to the 3′ end of amplified fragments, and signals of the methylated (M) and unmethylated (U) probes for RASSF1A CpG region #4 were shown, which reflected the indicated percentage of methylation. Lower panel, standard curve for measuring methylation level for RASSF1A CpG region #4 was shown. The intensity ratios (Y-axis) represented signal intensities of M/M+U. The linear distribution showed that measurements of the different mixtures were easily distinguished and used to determine the methylation level in the same CpG region for the patient samples. (B) Hybridization of three lung tumor samples to MSO microarray and images corresponding to the RASSF1A CpG region #4. Patient number 45 showed 0% methylation, but patient #16 and #9 displayed 58.4% and 79.3% methylation, respectively, based on the intensity ratios calculated from the standard curve. (0.09 MB DOC) [file pone.0012847.s001.doc]

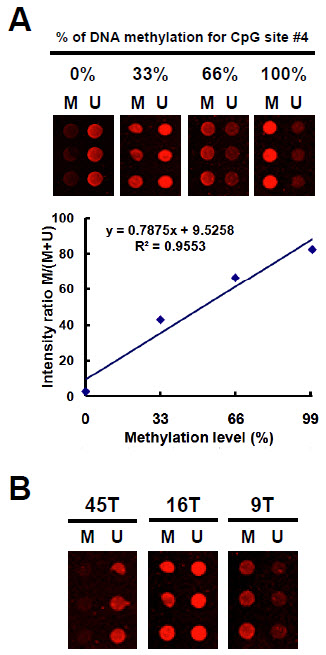


Figure S1. Representative MSO array figures. (A) Standardization curve for MSO assays. Upper panel, the series of MSO hybridization were performed with mixed samples containing 0, 33, 66, and 100% of *in vitro*-methylated DNA and amplified by PCR for the *RASSF1A* CpG island. The Cy5 fluorescent dye was added to the 3’ end of amplified fragments, and signals of the methylated (M) and unmethylated (U) probes for *RASSF1A* CpG region #4 were shown, which reflected the indicated percentage of methylation. Lower panel, standard curve for measuring methylation level for *RASSF1A* CpG region #4 was shown. The intensity ratios (Y-axis) represented signal intensities of M/M+U. The linear distribution showed that measurements of the different mixtures were easily distinguished and used to determine the methylation level in the same CpG region for the patient samples. (B) Hybridization of three lung tumor samples to MSO microarray and images corresponding to the *RASSF1A* CpG region #4. Patient number 45 showed 0% methylation, but patient #16 and #9 displayed 58.4% and 79.3% methylation, respectively, based on the intensity ratios calculated from the standard curve.
