# Supplementary material for: Distinct Epigenetic Domains Separated by a CTCF Bound Insulator between the Tandem Genes, BLU and RASSF1A
Source: PLoS One. 2010 Sep 20;5(9):e12847. doi: 10.1371/journal.pone.0012847 (PMC2942851; doi:10.1371/journal.pone.0012847)
Supplement: Figure S2 — Concordance analysis between mRNA expression (qRT-PCR) and methylation status (MSP) of RASSF1A and BLU genes. Y-axis represents the percentage of cases; X-axis represents the type of comparison. “+” indicates positive mRNA expression and DNA hypermethylation, as opposed to “−”, which indicates a negative result. Numbers above the bars indicate the percentage in the total concordant group (gray column) and discordant group (white column). P values are as indicated. (0.06 MB DOC) [file pone.0012847.s002.doc]

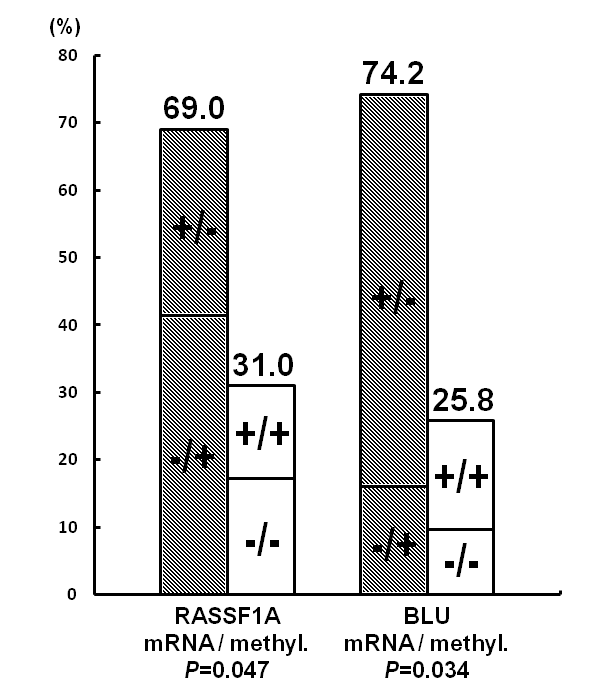


**Figure S2. Concordance analysis between mRNA expression (qRT-PCR) and methylation status (MSP) of *RASSF1A* and *BLU* genes.** Y-axis represents the percentage of cases; X-axis represents the type of comparison. “+” indicates positive mRNA expression and DNA hypermethylation, as opposed to “–”, which indicates a negative result. Numbers above the bars indicate the percentage in the total concordant group (gray column) and discordant group (white column). *P* values are as indicated.
