# Supplementary material for: Distinct Epigenetic Domains Separated by a CTCF Bound Insulator between the Tandem Genes, BLU and RASSF1A
Source: PLoS One. 2010 Sep 20;5(9):e12847. doi: 10.1371/journal.pone.0012847 (PMC2942851; doi:10.1371/journal.pone.0012847)
Supplement: Figure S3 — Three CTCF binding sites between RASSF1A and BLU genes by EMSA analysis. Three oligonucleotide probes (1, 2, and 3) containing putative CTCF binding sites in the BLU gene were synthesized and used for EMSA analysis. The biotin-labeled (hot probes) wild-type (Wt) oligonucleotide fragments were incubated with A549 nuclear extract and electrophoresed on 4% polyacrylamide gel. In the presence of anti-E2F1 antibody (lanes 1 to 3) and anti-IgG antibody (lanes 4 to 6), no super-shift complex was formed. The human apoB gene (lane 7) was the positive control for CTCF binding. Arrows indicated the band shift of specific protein-DNA complexes. The sequence information for EMSA probes is given in Table S1. (0.17 MB DOC) [file pone.0012847.s003.doc]

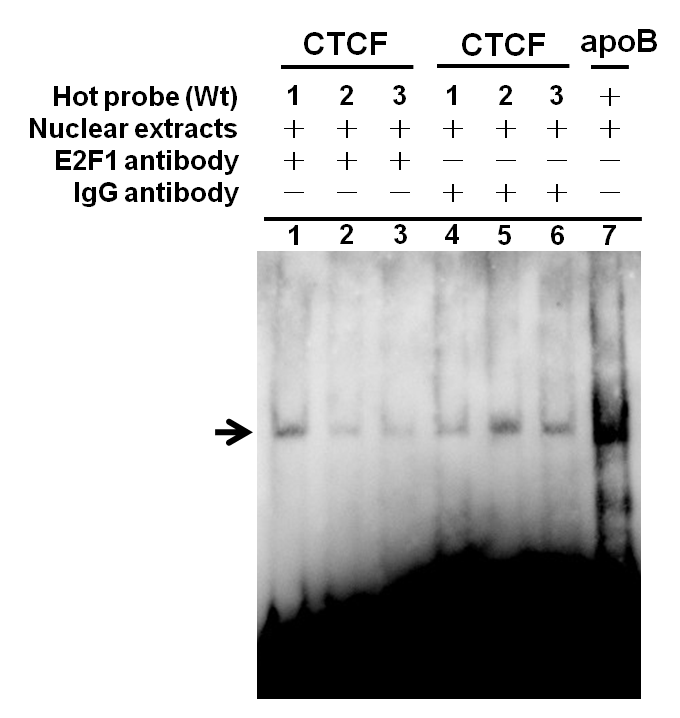


**Figure S3. Three CTCF binding sites between *RASSF1A* and *BLU* genes by EMSA analysis.** Three oligonucleotide probes (1, 2, and 3) containing putative CTCF binding sites in the *BLU* gene were synthesized and used for EMSA analysis. The biotin-labeled (hot probes) wild-type (Wt) oligonucleotide fragments were incubated with A549 nuclear extract and electrophoresed on 4% polyacrylamide gel. In the presence of anti-E2F1 antibody (lanes 1 to 3) and anti-IgG antibody (lanes 4 to 6), no super-shift complex was formed. The human *apoB* gene (lane 7) was the positive control for CTCF binding. Arrows indicated the band shift of specific protein–DNA complexes. The sequence information for EMSA probes is given in **Table S1**.
