# Supplementary material for: Distinct Epigenetic Domains Separated by a CTCF Bound Insulator between the Tandem Genes, BLU and RASSF1A
Source: PLoS One. 2010 Sep 20;5(9):e12847. doi: 10.1371/journal.pone.0012847 (PMC2942851; doi:10.1371/journal.pone.0012847)
Supplement: Figure S4 — CTCF binding assay and promoter methylation assay of RASSF1A and BLU genes in A549 and CL1-0 cancer cell lines. (A) ChIP-PCR assay for CTCF binding between RASSF1A and BLU genes in A549 and CL1-0 cell lines. “In”, total input DNA; “CTCF”, DNA-protein complex pulled down by anti-CTCF; “IgG”, DNA-protein complex pulled down with rabbit IgG; and “No”, no antibody. c-Myc served as a positive control for CTCF binding. D3D1568 microsatellite sequence served as a negative control for CTCF binding. (B) Methylation status of RASSF1A and BLU genes were assessed by MSP in MRC5 normal cell, A549 and CL1-0 cancer cell lines. U: unmethylated gene; M: methylated gene. SssI methyltransferase-treated MRC5 DNA was used as methylation positive control. (0.16 MB DOC) [file pone.0012847.s004.doc]

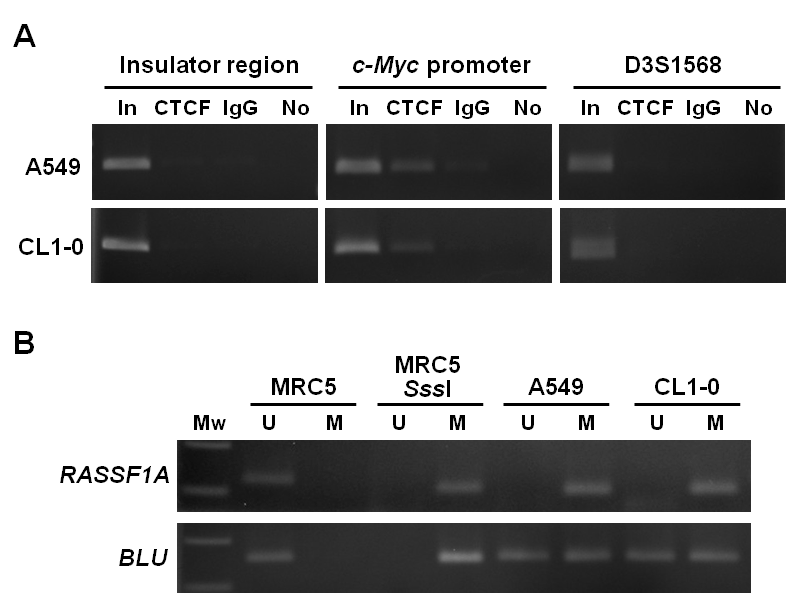


**Figure S4. CTCF binding assay and promoter methylation assay of *RASSF1A* and *BLU* genes in A549 and CL1-0 cancer cell lines. (A)** ChIP-PCR assay for CTCF binding between RASSF1A and *BLU* genes in A549 and CL1-0 cell lines. “In”, total input DNA; “CTCF”, DNA-protein complex pulled down by anti-CTCF; “IgG”, DNA-protein complex pulled down with rabbit IgG; and “No”, no antibody. *c-Myc* served as a positive control for CTCF binding. D3D1568 microsatellite sequence served as a negative control for CTCF binding. **(B)** Methylation status of *RASSF1A* and *BLU* genes were assessed by MSP in MRC5 normal cell, A549 and CL1-0 cancer cell lines. U: unmethylated gene; M: methylated gene. *Sss*I methyltransferase-treated MRC5 DNA was used as methylation positive control.
