# Supplementary material for: Distinct Epigenetic Domains Separated by a CTCF Bound Insulator between the Tandem Genes, BLU and RASSF1A
Source: PLoS One. 2010 Sep 20;5(9):e12847. doi: 10.1371/journal.pone.0012847 (PMC2942851; doi:10.1371/journal.pone.0012847)
Supplement: Figure S5 — Correlation analysis of mRNA and protein expression between RASSF1A and BLU. Y-axis: the percentage of cases; X-axis: the type of comparison. “+” indicated positive mRNA expression and protein expression, as opposed to “−”, which indicated a negative result. Numbers above the bars indicate the percentage in the total concordant group (gray column) and discordant group (white column). P values were compared results of RASSF1A with BLU. (0.05 MB DOC) [file pone.0012847.s005.doc]

**Figure S5. Correlation analysis of mRNA and protein expression between RASSF1A and BLU.** Y-axis: the percentage of cases; X-axis: the type of comparison. “+” indicated positive mRNA expression and protein expression, as opposed to “–”, which indicated a negative result. Numbers above the bars indicate the percentage in the total concordant group (gray column) and discordant group (white column). *P* values were compared results of RASSF1A with BLU.
