# Supplementary material for: Distinct Epigenetic Domains Separated by a CTCF Bound Insulator between the Tandem Genes, BLU and RASSF1A
Source: PLoS One. 2010 Sep 20;5(9):e12847. doi: 10.1371/journal.pone.0012847 (PMC2942851; doi:10.1371/journal.pone.0012847)
Supplement: Table S1 — Primer sequences, annealing temperature, and number of cycles in PCR reaction. (0.11 MB DOC) [file pone.0012847.s006.doc]

**Table S1: Primer sequences, annealing temperature, and number of cycles in PCR reaction.**

|  | **Primer name** | **Sequence (5’ to 3’)** | **Tm (^o^C)** | **PCR cycle no.** |
| --- | --- | --- | --- | --- |
| **RT-PCR** | | | | |
| RASSF1A | RASSF1A-F | GGCGTCGTGCGCAAAGGCC | 65 | 32 |
|  | RASSF1A-R | GGGTGGCTTCTTGCTGGAGGG |
| BLU | BLU-F | AACCAGCAGCATGAGAACCT | 65 | 32 |
|  | BLU-R | AGTTTGCGGTGGCAATAGT C |
| GAPDH | GAPDH-F | AATCCCATCACCATCTTC CA | 65 | 32 |
|  | GAPDH-R | CCTGCTTCACCACCTTCTTG |
| **qRT-PCR** | | | | |
| RASSF1A | qRASSF1A-F | TGCGCGCATTGCAAGTTC | 59 |  |
|  | qRASSF1A-R | TGGGCAGGTAAAAGGAAGTG |
| BLU | qBLU-F | AACCAGCAGCATGAGAACCT | 60 |  |
|  | qBLU-R | AGTTTGCGGTGGCAATAGT C |
| GAPDH | qGAPDH-F | GAGTCAACGGATTTGGTCGT | 58 |  |
|  | qGAPDH-R | TTGATTTTGGAGGGATCTCG |
| **MSO bisulfite-PCR** | | | | |
| RASSF1A | BT-P1-F | GTAGGTTAAGTGTGTTGTTTTAGT | 58 | 35 |
|  | BT-P1-R | TTACCCTTCCTTCCCTCCTT |
|  | BT-P2-F | AGGAGGGAAGGAAGGGTAAG | 56 | 35 |
|  | BT-P2-R | TAACTTTAAACRCTAACAAAC* |
|  | BT-E1-F | AAGTYGGGGTTYGTTTTGTGGTTT* | 58 | 35 |
|  | BT-E1-R | CCCCAAATAAAATCRCCACAAAAAT* |
| BLU | BT-P1-F | AGGATTTGGAGTTTAGGAGAGAT | 55 | 35 |
|  | BT-P1-R | CAATTCTCRCAACCATAAAAA* |
|  | BT-E1-F | TGTTTTGTTTTAGATTTTGGTAT | 52 | 35 |
|  | BT-E1-R | CCCRACTCAAAAACAATAACT* |
| **MSP** | | | | |
| RASSF1A | MSP1-U-F | TGGTTGTTGTTGTTGTGGTTGTTTG | 65 | 40 |
|  | MSP1-U-R | AAAACTATAAAACCCAAAAACAAAACTAAACACA |
|  | MSP1-M-F | GTCGTCGTTGTGGTCGTTC | 62 | 40 |
|  | MSP1-M-R | CCCGAAAACGAAACTAAACGCG |
|  | MSP2-U-F | TTTATTGTGTGGTTTTTTTTAGTTTTTTTTTGTTG | 65 | 40 |
|  | MSP2-U-R | TAACTTTAAACACTAACAAACACAAACCAAACA |
|  | MSP2-M-F | CGCGGTTTTTTTTAGTTTTTTTTCGTC | 65 | 40 |
|  | MSP2-M-R | TAACAAACGCGAACCGAACG |
| BLU | MSP-U-F | TTTGTGGGTTATAGTTTGAGAAAGTG | 62 | 38 |
|  | MSP-U-R | AACAAATTAACCACACCTACAC |
|  | MSP-M-F | TTCGTGGGTTATAGTTCGAGAAAGCG | 62 | 38 |
|  | MSP-M-R | AACGAATTAACCGCGCCTACGC |
| **Bisulfite-sequencing** | | | | |
| CTCF region | BS-P1-F | GTTAGGAAGGATATTTATGGGTTTG | 61 | 40 |
|  | BS-P1-R | TAAACCACCAACAAAATCAATTTAC |
|  | BS-P2-F | TTTTATGTAGGTGGGTTGAGGTTAT | 61 | 40 |
|  | BS-P2-R | CCAAAAACTATACTCCAACAATTCC |
|  | BS-P3-F | TTTAGGGTGATAGAGTTAAATGAGG | 61 | 40 |
|  | BS-P3-R | CCTCCCCCAAAATCCAAACTAAAC |
|  | BS-P4-F | TTTTATTTAGTGGGTAGGTTAAGTGTGTTG | 61 | 40 |
|  | BS-P4-R | CAACTCAATAAACTCAAACTCCCCC |
| **ChIP-PCR** | | | | |
| RASSF1A | ChIP-F | GTGGGGACCCTCTTCCTCTA | 68 | 40 |
|  | ChIP-R | GTGCTTCGCTGGCTTTGG |
| BLU | ChIP-F | CGCGAGAAATAGAGGAACCA | 68 | 40 |
|  | ChIP-R | GTCTGGGACAGGACAGTTGC |
| c-Myc | c-Myc-CTCF-F | GCCATTACCGGTTCTCCATA | 61 | 38 |
|  | c-Myc-CTCF-R | CAGGCGGTTCCTTAAAACAA |
| CTCF | ChIP-CTCF-F | TGGATGTGCTAGAGGCAGTG | 67 | 40 |
|  | ChIP-CTCF-R | CTGGACCGTGATCTTGAGGT |
|  | CTCF-Up-F | AGACTATTGCCACCGCAAAC | 70 | 35 |
|  | CTCF-Up-R | TGCTGAGAGAGAGGCTAGGG |
|  | CTCF-down-F | ACCTCAAGATCACGGTCCAG | 70 | 35 |
|  | CTCF-down-R | AATGGAAACCTGGGTGCAG |
|  | D3S1568-F | CCATGAACAGAACCTCCCTA | 65 | 35 |
|  | D3S1568-R | CCGCTGTCCTGCTGTAAG |
| **Luciferase reporter assay** | | | | |
| RASSF1A | Promoter-F | tccggtaccTGGAGAGCAGAGCGGGCGGTA^#^ | 65 | 40 |
|  | Promoter-R | ttcaagcttTCAGGCTCCCCCGACATGGC^#^ |
|  | E2F1-Mut-1-F | CGCGTGGTGCTTTGATTTCGCCGTCGTTGTGG^†^ | 70 | 18 |
|  | E2F1-Mut-1-R | CCACAACGACGGCGAAATCAAAGCACCACGCG^†^ |
|  | E2F1-Mut-2-F | CCCAGGTTTCCATTGCGATTCTCTCCTCAGCTCC^†^ | 70 | 18 |
|  | E2F1-Mut-2-R | GGAGCTGAGGAGAGAATCGCAATGGAAACCTGGG^†^ |
|  | E2F1-Mut-3-F | CTCTCCTCAGCTCCTTCAATACGCCCAGTCTGGATCCTG^†^ | 70 | 18 |
|  | E2F1-Mut-3-R | CAGGATCCAGACTGGGCGTATTGAAGGAGCTGAGGAGAG^†^ |
| **EMSA** | | | | |
| apoB | apoB-CTCF-S | CAAATTATCCTGCCCCCTAGACATAACCTCCC |  |  |
|  | apoB-CTCF-AS | GGGAGGTTATGTCTAGGGGGCAGGATAATTTG |  |  |
| CTCF-1 | CTCF(EMSA)-1-S | CTGCCCTGCCTCCTGGTGGAACTGCTG |  |  |
|  | CTCF(EMSA)-1-AS | CAGCAGTTCCACCAGGAGGCAGGGCAG |  |  |
|  | CTCF(EMSA)-1(mut)-S | TTGCCATGCTTCAAGGTGGAACTAATG |  |  |
|  | CTCF(EMSA)-1(mut)-AS | CATTAGTTCCACCTTGAAGCATGGCAA |  |  |
| CTCF-2 | CTCF(EMSA)-2-S | TCACACTGGCCTCCACTGGACACCC |  |  |
|  | CTCF(EMSA)-2-AS | GGGTGTCCAGTGGAGGCCAGTGTGA |  |  |
|  | CTCF(EMSA)-2(mut)-S | TCACATTGGCATGAAGTGGACACCC |  |  |
|  | CTCF(EMSA)-2(mut)-AS | GGGTGTCCACTTCATGCCAATGTGA |  |  |
| CTCF-3 | CTCF(EMSA)-3-S | CCCACCTGCCTGCAGGGAGTGCCAAG |  |  |
|  | CTCF(EMSA)-3-AS | CTTGGCACTCCCTGCAGGCAGGTGGG |  |  |
|  | CTCF(EMSA)-3(mut)-S | CCTACAAGCGAGTAGGGAATGCCAAG |  |  |
|  | CTCF(EMSA)-3(mut)-AS | CTTGGCATTCCCTACTCGCTTGTAGG |  |  |

* R: mixture of A and G; Y: mixture of G and T.

^#^ the restriction enzyme digestion site is shown in lower case letters.

^†^ Underlined sequences were mutated in E2F1 binding sites.
